# Investigation on Precursor Aromas and Volatile Compounds During the Fermentation of Blackened Pear Vinegar

**DOI:** 10.3390/foods14162905

**Published:** 2025-08-21

**Authors:** Shangjing Chen, Yuxiao Wang, Xin Sun, Zhizhen Han, Qiyong Jiang, Lin Gao, Rentang Zhang

**Affiliations:** 1 College of Food Science and Engineering, Shandong Agricultural University, Tai’an 271018, China; 2 Management Service Center of Laoling Agricultural Hi-Tech Industries Demonstration Zone, Dezhou 253600, China; 3 Laoling Healthy Food Industry Technology Research Institute, Dezhou 253600, China; 4 Laoling Tailetang Food Technology Co., Ltd., Dezhou 253600, China

**Keywords:** backened pear vinegar, flavor compounds, organic acids, free amino acids, dynamic changes

## Abstract

The acetic acid fermentation stage is a key determinant of fruit vinegar’s aroma profile. Therefore, this study employed GC-MS, HPLC, E-nose and E-tongue techniques, in conjunction with multivariate statistical analysis, to investigate the dynamic changes of compounds during the acetic acid fermentation process of blackened pear vinegar (BPV), as well as the transformation of volatile and non-volatile aroma-active compounds. Results revealed accumulation of organic acids and esters alongside declines in alcohols, aldehydes, and ketones. Isoamyl acetate, benzaldehyde, and nonanal (OAV > 1) were identified as key aroma contributors (VIP > 1, *p* < 0.05). Total organic acids significantly increased from 4.82 ± 0.53 mg/mL to 10.29 ± 2.38 mg/mL. Correlation analysis revealed a negative relationship between amino acids and volatile compounds, and this negative correlation suggests a possible precursor–product relationship between them. These findings provide theoretical support for the enhancement of fruit vinegar flavor, as well as the application of blackened fruits.

## 1. Introduction

Pear (*Pyrus* spp.), a member of the Rosaceae family, exhibits rich germplasm diversity and a long-standing cultivation history [1]. It serves as a rich source of dietary fiber, sugars, bioactive components, and vitamins [2]. Research has shown that the blackening process significantly enhances the antioxidant activity and the content of bioactive substances, including total phenolic contents (TPC) and total flavonoid contents (TFC) in jujube, thereby improving its nutritional value [3,4]. The blackening process boosts the levels of bioactive compounds like anthocyanins and polyphenols in garlic, while reducing off-flavors, thus improving its nutritional value and edibility [5]. It was reported that macromolecular degradation, chemical oxidation, and non-enzymatic browning reaction were responsible for fruit blackening [6]. While blackening technology has been widely applied, further research is required to explore its derivative products and optimize their functional properties. Therefore, using blackened pears as a raw material for further processing is a promising choice.

Vinegar, particularly fruit-based varieties, has gained significant attention in recent years as a functional fermented beverage [7]. It offers lipid-lowering effects, antimicrobial activity, anticancer potential, antioxidant, and antiaging properties [8]. The sensory profile of vinegar, particularly its flavor characteristics, serves as a key determinant of product quality and plays a pivotal role in consumer preference. Interestingly, the variation in flavor compounds is complex due to the multiple biochemical reactions occurring during fermentation [9]. The flavor profile and nutritional enhancement of fruit vinegars are significantly influenced by their organic acids and free amino acid composition [10,11]. Investigations reveal an association between organic acids and distinct aromatic compounds [12]. Therefore, it is crucial to elucidate the dynamic evolution patterns of flavor precursors and volatile flavor compounds during vinegar fermentation, and their interrelationship.

Although previous studies have explored blackened fruits and various fruit vinegars, the dynamic changes and transformations of compounds during the acetic acid fermentation process of blackened fruit vinegar are not yet well understood. Therefore, this study utilized blackened pears as the raw material and comprehensively monitored the changes in key physicochemical parameters during the fermentation of BPV, including TPC, TFC, polysaccharide concentration, and 5-hydroxymethylfurfural (5-HMF). GC-MS, HPLC, E-nose and E-tongue, coupled with multivariate statistical analysis, were employed to investigate the dynamic profiles of both nonvolatile flavor compounds (organic acids and free amino acids) and volatile flavor compounds, and a unique precursor–product conversion network for BPV was established. These findings will provide a scientific foundation for expanding the potential applications of blackening fruits and achieving targeted aroma modulation.

## 2. Materials and Methods

### 2.1. Materials and Reagents

Fresh pears (*Pyrus* spp.) were purchased from the market (Laiwu, Jinan, China). *Acetobacter pasteurianus* CICC 20001 was acquired from Shanghai Jiamin Fermentation Food Co., Ltd. (Shanghai, China). The amino acids mixture standard solution (Type H) was obtained from Wako Pure Chemical Industries, Ltd. (Osaka, Japan). Standards for the determination of HPLC, including citric acid (99.5%), malic acid (98%), lactic acid (98%), succinic acid (99%), tartaric acid (99%), fumaric acid (98%), quinic acid (98%), and acetic acid (99.7%) were purchased from Shanghai yuanye Bio-Technology Co., Ltd. (Shanghai, China). 2-octanol (99.5%, GC-Grade), sodium chloride, H_3_PO_4_ (HPLC-Grade), methanol (HPLC-Grade), 5-HMF (99%, HPLC-Grade), glucose, phenol, sulfuric acid, rutin, sodium nitrite, aluminum nitrate, gallic acid, Folin–Ciocalteu reagent, and sodium carbonate were purchased from Macklin Biochemical Co., Ltd. (Shanghai, China). Unless indicated, the reagents and chemicals utilized in this study were of analytical reagent grade.

### 2.2. Preparation of BPV

The preparation of blackened pear was based on methods described in previous studies with slight modifications [6]. The pear slices were dehydrated in a convection oven at 70 °C until a final moisture content of 35% was achieved, followed by sealed blackening treatment under controlled conditions (70 °C, 35% relative humidity) for six days. The blackened pears were mixed with water at a ratio of 350 g/L, followed by juicing and filtration to obtain clarified blackened pear juice. The juice was then heated at 65 °C for 30 min, cooled, and food-grade ethanol was added to adjust the initial alcohol concentration to 9% (*v*/*v*). Subsequently, the activated *Acetobacter pasteurianus* CICC 20001 (10%) was inoculated into the mixture, and the fermentation was carried out aerobically at 30 °C in a controlled incubator for 10 days [13]. Samples of BPV were collected at designated time intervals (days 0, 2, 4, 6, 8, and 10) during the 10-day fermentation process.

### 2.3. Determination of TPC, TFC, Polysaccharides, and 5-HMF During the Fermentation of BPV

TPC was quantified using spectrophotometric analysis with the Folin–Ciocalteu method [14]. TFC was measured by means of the colorimetric assay technique [15]. Prior to TPC measurement, 4 mL of 80% ethanol was added to 1 mL of the sample for polyphenol extraction, followed by a 5-fold dilution. After centrifugation, the subsequent steps were performed using the Folin–Ciocalteu colorimetric method. Similarly, before TFC determination, the sample was diluted 10 times before proceeding with the subsequent experiments. The polysaccharide content was determined by the phenol–sulfuric acid assay [13].

The concentration of 5-HMF was quantified using HPLC [3]. Briefly, BPV (5 mL) was collected and centrifuged for 10 min, and filtration of the supernatant was carried out. The 2:98 (*v*/*v*) methanol–water mixture was used as the mobile phase, and detection was carried out at 282 nm using HPLC (LC-20A, Shimadzu, Kyoto, Japan). Chromatographic separation was performed using an IntertSustain C18 column (250 mm × 4.6 mm, 5 μm; Shimadzu, Kyoto, Japan) maintained at 35 °C.

### 2.4. Quantitative Analysis of Organic Acids by HPLC

The quantification of organic acids was conducted via HPLC. Briefly, the fermented sample (1 mL) was subjected to centrifugation at 8000× *g* for 5 min. The HPLC system (LC-20A, Shimadzu, Kyoto, Japan) was outfitted with an AQ-C18 column (4.6 mm × 150 mm, 5 μm; Welch Materials, Shanghai, China). Chromatographic separation was conducted at 40 °C with 0.01 mol/L H_3_PO_4_ serving as the mobile phase, and detection at 210 nm [7].

### 2.5. Analysis of Free Amino Acids by Amino Acid Analyzer

Free amino acid quantification was carried out using an automated amino acid analyzer (LA-8080, Hitachi, Tokyo, Japan), following the method with slight modifications [16]. In brief, samples (1 mL) were placed into 10 mL volumetric flasks and extracted with 0.02 mol/L hydrochloride. Following the addition of an equivalent volume of a 5% sulfosalicylic acid solution, the mixture was incubated for 15 min. Subsequently, centrifugation was conducted at 8000× *g* for 15 min. The supernatant was separated by ion-exchange chromatography using a 4.6 mm ID × 60 mm column, and amino acids were detected via the ninhydrin reaction.

### 2.6. Determination of Volatile Flavor Compounds

HS-SPME-GC–MS was used to examine the aroma components of the vinegar [10]. After adding 5 mL of the sample and 1 g of sodium chloride to a 20 mL vial, the 10 μL of 2-octanol (internal standard, 0.16 mg/L) dissolved in absolute methanol was introduced. Volatile compounds were extracted using a 50/30 μm DVB/CAR/PDMS SPME fiber (Supelco, Bellefonte, PA, USA), which was inserted into the vial headspace. The vial was tightly sealed with a silicone septum and incubated in a 40 °C water bath for 30 min. Following the extraction, the fiber was removed and directly inserted into the GC inlet (set to 240 °C) for desorption, which lasted for 5 min. Using a DB-WAX column (Agilent, Santa Clara, CA, USA), volatile components were separated. The initial temperature was set at 40 °C for 10 min, raised to 140 °C (at 5 °C/min), then increased to 230 °C at 10 °C/min. Unidentified compounds were characterized by mass spectrometry and matched to the NIST 14 library based on retention time. The relative concentrations of volatile compounds were determined by comparing the chromatographic peak areas of volatile flavor compounds with those of the internal standard. The calculation formula is as follows: C = A × Ci/Ai [10,17]. Where A is the chromatographic peak area of the volatile compound; Ci is the concentration of the internal standard 2-octanol, μg/L; Ai is the chromatographic peak area of 2-octanol; and C represents the concentration of the volatile compound, μg/L.

### 2.7. Analysis of Characteristic Flavor Compounds Based on Odor Activity Value (OAV)

The distinctive flavor components in each fermentation stage were identified using the OAV. Compounds might be recognized as typical flavor compounds when their OAV was more than or equal to 1, where OAV = mass concentration/odor threshold [11].

### 2.8. E-Nose and E-Tongue Analysis of BPV

The odor of the sample was evaluated with the Airsence Pen3 Electronic Nose System (Airsence, Schwerin, Germany). For headspace enrichment, the samples were incubated in a water bath at 40 °C for 30 min before analysis. The main applications of the sensors are shown in Table 1. The parameters of the E-nose were as follows: sampling flow 350 mL/min, sample interval time 120 s, probe insertion time 5 s, balance time 10 s, and sampling time 120 s [6]. The taste of BPV was analyzed using an SA402B Plus-EX system (Instent, Yokohama, Japan). After centrifuging the sample at 9000× *g* for 8 min, it was diluted 25-fold and then analyzed (70 mL).

### 2.9. Statistical Analysis

Bar charts and scatter plots were generated using GraphPad Prism (version 9.0, GraphPad Software, San Diego, CA, USA). Multivariate statistical analysis, including variable importance in projection (VIP) calculation, partial least squares discriminant analysis (PLS-DA), and orthogonal partial least squares discriminant analysis (OPLS-DA), was conducted using SIMCA software (Version 14.1, Umetrics, Umea, Sweden). Heatmaps were generated using Origin 2021 software and https://www.omicstudio.cn/tool (accessed on 19 February 2025). The correlation network was constructed by Cytoscape v.3.9. Significant differences between groups were evaluated using an ANOVA with SPSS (Version 26.0, IBM SPSS Statistics, Armonk, NY, USA) and were deemed statistically significant at the *p* < 0.05 level. All experiments were conducted in three independent trials, and the mean ± standard deviation (SD) was used to express the results. Due to significant variability in amino acid measurements, five experimental replicates were performed to ensure data reliability.

## 3. Results and Discussion

### 3.1. Analysis of Morphological and Physicochemical Properties

**Figure 1:**
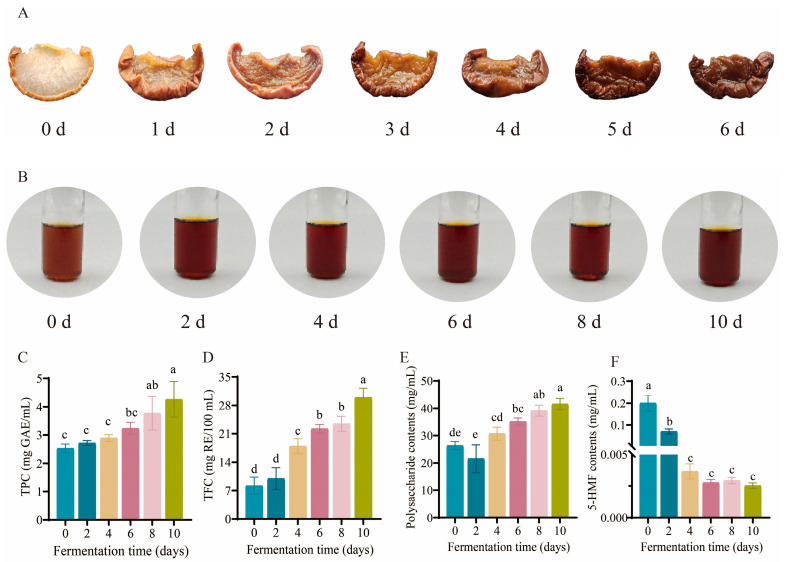
Analysis of morphological and physicochemical properties. Results show (**A**) morphological changes during pear blackening, (**B**) appearance changes during the fermentation of BPV, (**C**) dynamic profiles of TPC, (**D**) temporal variation in TFC, (**E**) polysaccharide content variation, and (**F**) evolution of 5-HMF levels Bars labeled with different lowercase letters are significantly different (*p* < 0.05).

The morphological changes in pears during blackening are shown in Figure 1A, while Figure 1B presents the alterations in BPV across fermentation stages. The dynamic trends of bioactive components during BPV fermentation are illustrated in Figure 1C–F and Table S1. Phenolic compounds, a prominent group of secondary plant metabolites, are widely recognized for their antioxidant activity [18]. As illustrated in Figure 1C, the TPC in BPV increased significantly from 2.53 ± 0.16 mg GAE/mL on day 0 to 4.26 ± 0.63 mg GAE/mL by day 10. The increase in TPC may be due to the microbial metabolic activity during fermentation, which promotes the release of bound phenolic compounds [14,19].

As shown in Figure 1D, the TFC of BPV increased progressively during fermentation, rising from 8.17 ± 2.11 mg RE/100 mL on day 0 to 29.94 ± 2.29 mg RE/100 mL by day 10. This increase is attributed to a synergistic effect of enzymatic degradation of cell wall components and microbial metabolism [20]. The dynamic changes in polysaccharide content during BPV fermentation are depicted in Figure 1E. Initially, polysaccharide concentration decreased from 26.34 ± 1.49 mg/mL on day 0 to 21.58 ± 5.04 mg/mL by day 2, followed by a subsequent increase to 41.56 ± 2.07 mg/mL on day 10. During early fermentation, microbial hydrolytic enzymes break glycosidic bonds in polysaccharides, leading to depolymerization into oligosaccharides and monosaccharides, which results in a reduction in polysaccharide content [18]. The subsequent increase in polysaccharide levels could be due to the microbial synthesis of exopolysaccharides [21], such as levan, which can be produced by *Acetobacter* [22]. It is therefore plausible that such exopolysaccharides may also be synthesized during the fermentation process of BPV. Additionally, the cell wall structure of the raw material is decomposed by the action of microorganisms and enzymes, leading to the release of polysaccharides previously bound within the cell wall into the fermentation liquid [23]. Insoluble polysaccharides can also be gradually degraded into soluble polysaccharides under the action of relevant enzymes, leading to an increase in the polysaccharide content in the fermentation liquid [24]. A similar trend has been observed in the fermentation of wolfberry vinegar [15].

5-HMF is a byproduct of the browning process, formed during thermal processing [5]. Non-enzymatic browning during the blackening process of fruits leads to an increase in 5-HMF content [4]. 5-HMF possesses antioxidant properties and promotes blood circulation; however, it is primarily metabolized in the human body into the toxic compound 5-sulfonylmethylfurfural (5-SMF), which has been linked to carcinogenesis and proximal renal tubular damage [25,26]. As shown in Figure 1F, the initial concentration of 5-HMF in BPV was 0.2003 ± 0.0371 mg/mL, and it decreased sharply, dropping to 0.0029 ± 0.0003 mg/mL by day 8 and further declining to 0.0025 ± 0.0002 mg/mL by the conclusion of fermentation. This procedure effectively degraded the furfural compounds generated during thermal treatment, thereby reducing the intake of 5-HMF.

### 3.2. Dynamic Changes in Organic Acids

**Figure 2:**
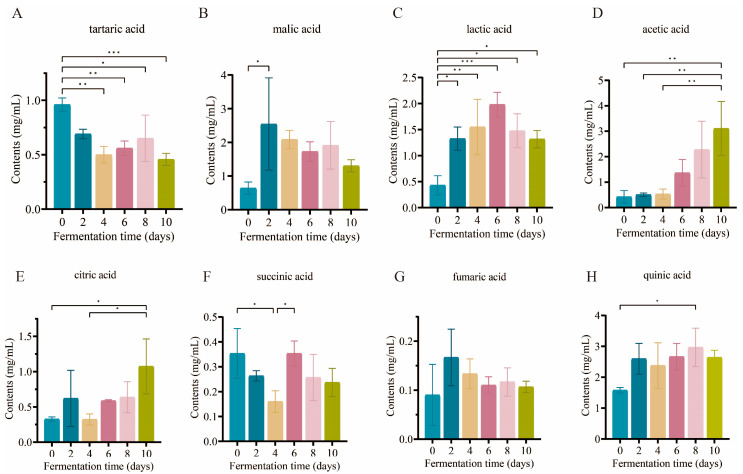
Dynamic trends of organic acids. Results show (**A**) acetic acid, (**B**) malic acid, (**C**) lactic acid, (**D**) tartaric acid, (**E**) citric acid, (**F**) succinic acid, (**G**) fumaric acid, and (**H**) quinic acid (* *p* < 0.05, ** *p* < 0.01, *** *p* < 0.001).

Organic acids serve as both bioactive components and nutrients, and also play a role in the development of vinegar flavor [8]. By using HPLC, eight organic acids from BPV samples at various phases of fermentation were quantitatively examined, including tartaric acid, malic acid, lactic acid, acetic acid, citric acid, succinic acid, fumaric acid, and quinic acid (Figure 2A–H and Table S2). The total organic acid content exhibited a continuous increase, reaching a peak concentration of 10.29 ± 2.38 mg/mL on day 8, after which it stabilized (Table S3). Predominant among the organic acids were acetic acid (Figure 2A) and quinic acid (Figure 2H), together comprising over 56% of the total organic acids.

During the fermentation, the content of acetic acid gradually increased, which is attributed to the oxidation of alcohols into acetic acid by *Acetobacter* [27]. The lactic acid content (Figure 2C) initially increased before subsequently decreasing. The initial accumulation of lactic acid is predominantly linked to the metabolic activity of lactic acid bacteria [27]. However, its subsequent decline is likely a result of the inhibitory impacts of accumulated acetic acid on the metabolic activity of these bacteria [28]. Malic acid (Figure 2B), citric acid (Figure 2E), succinic acid (Figure 2F), and fumaric acid (Figure 2G) are intermediate metabolites in the TCA cycle, synthesized and interconverted within the metabolic pathway [29]. During acetic acid fermentation, the enzymes of the TCA cycle in acetic acid bacteria are highly induced, which influences the generation of metabolic products [30]. Research has demonstrated that non-volatile acids are essential for moderating the sharp acidity imparted by acetic acid in vinegar [20].

### 3.3. Dynamic Variation in Free Amino Acids

**Figure 3:**
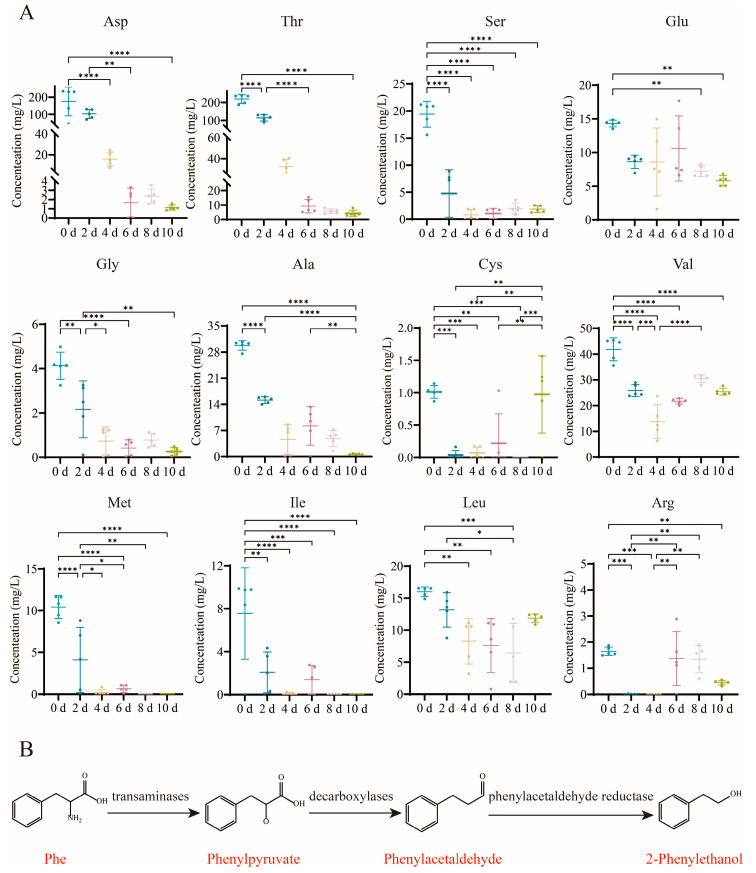
Dynamic trends of free amino acids. Results show (**A**) notable changes in the concentrations of 12 free amino acids during the fermentation of BPV (* *p* < 0.05, ** *p* < 0.01, *** *p* < 0.001, **** *p* < 0.0001), and (**B**) the Ehrlich pathway of free amino acids, taking Phe (phenylalanine) as an example.

Table S4 clearly demonstrates that certain free amino acids exhibited significant changes during fermentation, while others remained relatively stable. For clarity, we focused on displaying free amino acids with marked variations in Figure 3. During the fermentation of BPV, the concentrations of all amino acids exhibited dynamic changes corresponding to the fermentation progression.

The content of free amino acids exhibited a decreasing trend, declining from 561.16 ± 116.21 mg/L to 60.16 ± 2.46 mg/L. This result aligns with prior research [31]. Among the 17 free amino acids identified, glutamic acid (Glu), leucine (Leu), and valine (Val) were found to be the most abundant, collectively accounting for approximately 59% of the total free amino acids in the vinegar. Val had the highest concentration, which decreased from 41.82 ± 4.48 mg/L to 25.43 ± 1.37 mg/L during acetic acid fermentation. Leu and Glu followed, with Leu decreasing from 15.99 ± 0.74 mg/L to 11.86 ± 0.65 mg/L, and Glu reducing from 14.31 ± 0.50 mg/L to 5.80 ± 0.76 mg/L. One possible reason for the significant reduction in free amino acid content is their degradation into flavor compounds. For example, the formation of alcohols is associated with certain specific amino acids, which can be converted into alcohols via the Ehrlich pathway (Figure 3B) [32], such as valine (Val) to 2-methylpropanol, Phe to 2-phenylethanol, and leucine (Leu) to 3-methylbutanol [33]. Additionally, aspartic acid (Asp) can be converted into acetoin through a series of enzymatic reactions [34], which may be one of the potential reasons for the observed increase in acetoin content in this study (Table S5). Ketones and aldehydes are primarily generated through the degradation of amino acids [35]. Moreover, during acetic acid fermentation, free amino acids can be utilized as a nitrogen source by *Acetobacter* [36]. Although proteins in the raw materials are rapidly hydrolyzed by microorganisms and enzymes, leading to the formation of amino acids, the rate of amino acid consumption exceeded their synthesis, leading to a sustained decrease in amino acid levels [37].

### 3.4. Dynamic Changes in Volatile Flavor Compounds During the Fermentation of BPV Analyzed by HS-SPME-GC–MS and OAV

**Figure 4:**
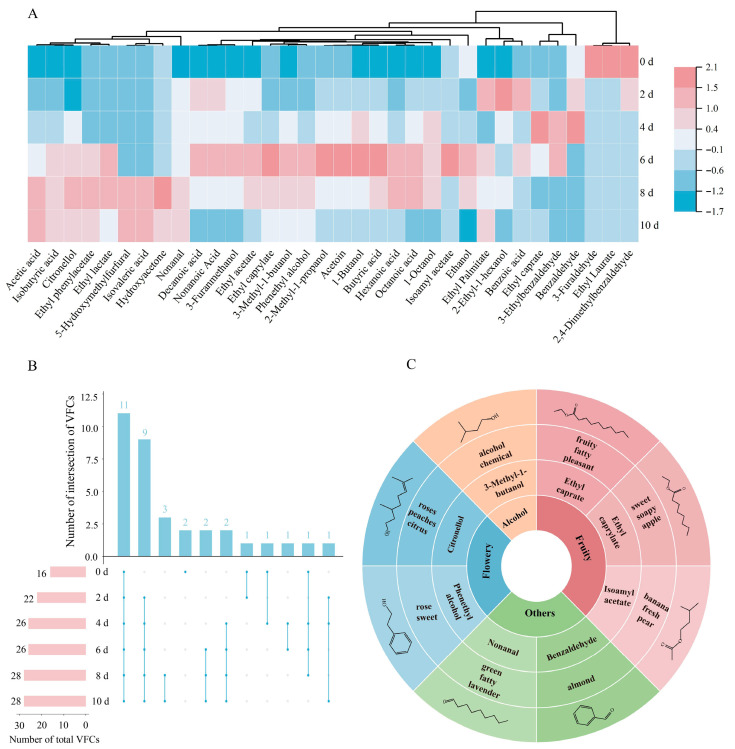
Dynamic evolution of volatile flavor compounds during the fermentation of BPV. Results show (**A**) heatmap of compound concentrations, with red indicating high concentration, white representing medium concentration, and blue denoting low concentration, (**B**) UpsetR diagram illustrating shared and unique flavor substances across fermentation stages (VFCs: volatile flavor compounds), and (**C**) flavor wheel of nine key odor characteristic components, displaying from innermost to outermost, including subjective sensory categories, characteristic compounds, associated odor descriptors, and chemical structures of characteristic compounds.

A total of 34 volatile flavor compounds were identified in samples during the fermentation process of BPV. As shown in Figure 4A and Table S5, the volatile flavor compounds comprised substances with varying types and concentrations. Fermentation significantly increased the concentration of volatile flavor compounds in BPV. Specifically, the total volatile concentration increased from 7237.80 ± 1348.16 μg/L on day 0 to 16,474.26 ± 1556.72 μg/L by day 10. As shown in Figure 4B, there were 28 volatile flavor compounds detected at the end of fermentation, compared to 16 compounds in the initial stage, with 12 newly emerged compounds added and 11 compounds persistently present throughout the fermentation process.

Beyond the ethanol fermentation substrate, BPV contained substantial concentrations of isoamyl alcohol and phenethyl alcohol in its volatile alcohol composition. Moderate levels of alcohols contribute to a more balanced and mellow flavor profile in vinegar [11]. Under aerobic conditions, acetic acid bacteria convert ethanol into acetic acid [38]. Consequently, ethanol declined progressively throughout fermentation, decreasing from 6452.31 ± 1285.54 μg/L on day 0 to 2432.45 ± 704.7 μg/L by day 10, representing a 62.3% reduction in ethanol content. The observed increase in isoamyl alcohol content could be attributed to microbial metabolism of residual substrates, potentially mediated through the Ehrlich pathway involving amino acid degradation [33]. Furthermore, the OAV of phenethyl alcohol exceeded 1, as shown in Table S6, suggesting its contribution to the rose-like and floral aroma characteristics of BPV [39].

Acid accumulation during fermentation is predominantly driven by the metabolic activity of acetic acid bacteria [40]. A total of nine volatile acids were identified in BPV, with a cumulative concentration of 11,955.45 ± 1095.56 μg/L at the end of fermentation. Among these, acetic acid was the predominant component, significantly influencing both the sour taste and the overall aromatic profile of the vinegar. Notably, acetic acid also serves as a key precursor in esterification reactions, and the increase in its concentration further enhances the aromatic complexity of the final product [41]. The majority of acids exhibit fatty, pungent, and fruity sensory attributes, which significantly influence the overall flavor profile of vinegar [10].

Esters primarily derive from the fruit itself and enzymatically catalyzed reactions between carboxylic acids and alcohols during fermentation [39]. In BPV, ester concentrations exhibited dynamic fluctuations throughout the fermentation process, rising from 64.56 ± 31.59 μg/L on day 0 to a peak of 2478.21 ± 1917.67 μg/L by day 6, before gradually declining thereafter. The decline in ester concentrations during the later stages of fermentation could be due to chemical degradation and volatilization losses [10]. In the initial stages of fermentation (day 0), a total of four esters were identified, whereas by day 10, seven esters were detected. Additionally, as shown in Table S6, ethyl caprate, ethyl caprylate, and isoamyl acetate in BPV at the final fermentation stage exhibited OAVs greater than 1, contributing to its characteristic fruity aroma.

The concentrations of aldehydes and ketones in BPV initially exhibited a significant increase. However, due to their intrinsic chemical instability, these compounds underwent oxidation to carboxylic acids, resulting in a progressive reduction in their levels [7]. 3-Hydroxy-2-butanone (acetoin), the primary ketone contributing to the buttery aroma of BPV, was generated via oxidation of 2,3-butanediol [41]. Its concentration increased from 0 μg/L to 204.73 ± 48.62 μg/L during fermentation, subsequently declining to 33.99 ± 13.42 μg/L by day 10. The aldehyde formation is closely associated with amino acids metabolism [42]. The concentration of benzaldehyde increased from 16.18 ± 3.06 μg/L to 33.52 ± 1.51 μg/L, which could be attributed to the decarboxylation and deamination of methionine and phenylalanine [10].

The threshold is typically defined as the minimum concentration of an aromatic compound required for human olfactory detection [10]. Compounds with higher OAV made more significant contributions to the overall flavor profile [11]. As shown in Figure 4C and Table S6, OAVs were conducted to characterize the dynamic changes in aroma. During the initial stages of fermentation, only ethyl caprylate (sweet, soapy, apple; 1.76) and benzaldehyde (green, fatty, lavender; 1.16) had OAVs greater than 1. In the later stages of fermentation, the most characteristic compounds in blackened pear vinegar were isoamyl acetate (banana, fresh, pear; 5.46), nonanal (almond; 7.25), and ethyl caprylate (sweet, soapy, apple; 6.73), followed by 3-Methyl-1-butanol (alcohol, chemical; 1.10) and citronellol (roses, peaches, citrus; 1.07). During the fermentation process, the characteristic fruity aroma intensifies rapidly.

### 3.5. Differences in Aroma Characteristics Analyzed by Multivariate Data Analysis

**Figure 5:**
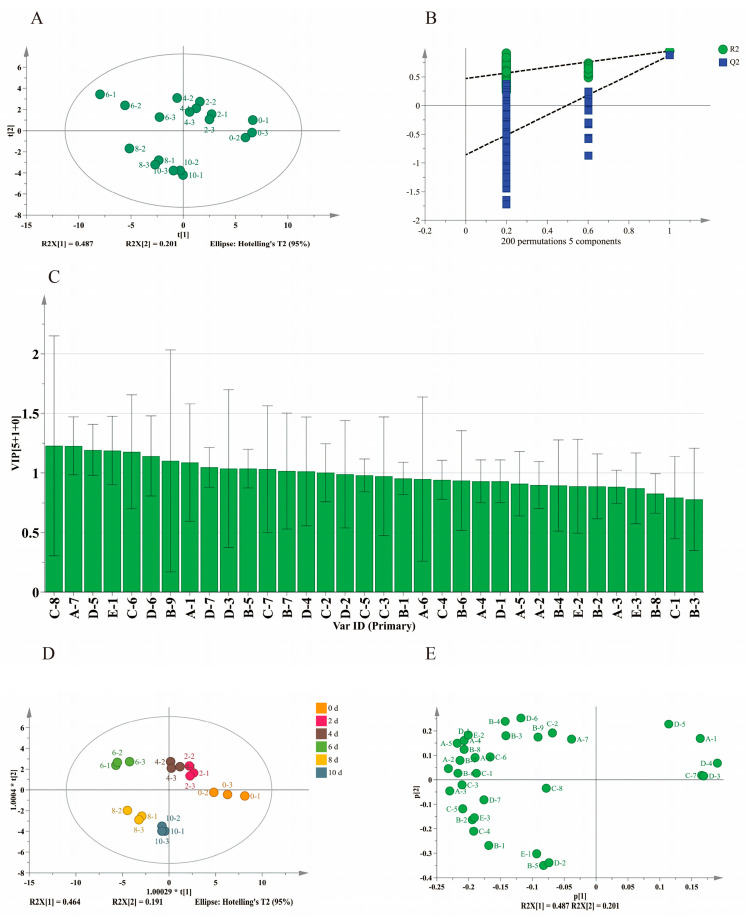
Multivariate statistical analysis of volatile flavor compounds during the fermentation of BPV. (**A**) Score plot for the PCA model, (**B**) loading plot for the PCA model, (**C**) VIP plot (VIP > 1), (**D**) score plot of the OPLS-DA, and (**E**) validation of OPLS-DA model. (Corresponding compounds: C-8 ethyl palmitate, A-7 2-ethyl-1-hexanol, D-5 benzaldehyde, E-1 hydroxyacetone, C-6 isoamyl acetate, D-6 3-ethylbenzaldehyde, B-9 benzoic acid, A-1 ethanol, D-7 nonanal, B-5 isovaleric acid, D-3 3-furaldehyde, C-7 ethyl laurate, D-4 2,4-dimethylbenzaldehyde, B-7 octanoic acid, D-2 5-hydroxymethylfurfural).

Figure 5A,B displays the PCA scores and loading plots. As illustrated in Figure 5A, the entire fermentation process of BPV could be classified into three distinct phases: Phase I (0–4 days), Phase II (6 days), and Phase III (8–10 days). The relative positioning of the samples reflects variations in their aromatic profiles. During fermentation, the samples shifted counterclockwise from the first quadrant (0–4 days) to the second quadrant (6 days), and ultimately to the third quadrant (8–10 days). Notably, aldehydes and alcohols were primarily located in the first quadrant, whereas acids and esters were predominantly distributed across the second and third quadrants (Figure 5B). This suggests that aldehydes and alcohols were mainly associated with the early and mid-fermentation stages (Phases I and II), with their concentrations decreasing over time. In contrast, acids and esters were more prevalent during the later stage (Phase III), indicating a transition in volatile compound composition as fermentation progressed. These observations align with the findings in Section 3.4 and Table S5.

The contribution of each variable was quantified by calculating VIP scores from the OPLS-DA model [9]. As shown in Figure 5D, the OPLS-DA score scatter plot demonstrated robust model performance, with R^2^X = 0.902, R^2^Y = 0.958, and Q^2^ = 0.819. The predictive parameter (Q^2^ = 0.819) and applicability parameter (R^2^Y = 0.958) confirmed the model’s reliability. The slopes of the R^2^ and Q^2^ regression lines were >0, while the intercept of the Q^2^ regression line was <0 (−0.817), collectively indicating no overfitting (Figure 5E). The OPLS-DA score plot effectively distinguished BPV samples from various fermentation stages, revealing distinct shifts in volatile flavor compounds between the early and late phases. However, samples from days 8 and 10 clustered together, suggesting minimal changes in volatile compounds during the later stages. This observation points to a gradual stabilization of the aromatic profile as fermentation progresses. As illustrated in Figure 5C, based on the VIP scores, 15 volatile flavor compounds in BPV were identified as significant markers (VIP > 1, *p* < 0.05), highlighting their critical roles in the overall flavor profile. These volatile markers can serve as critical indicators for evaluating the degree of fermentation in BPV. Notably, three volatile flavor compounds (isoamyl acetate, benzaldehyde, and nonanal) were identified as both key volatile flavor compounds (OAV > 1) and differential markers (VIP > 1, *p* < 0.05). Isoamyl acetate emerged on day 6, benzaldehyde was gradually depleted, and nonanal increased progressively throughout fermentation.

### 3.6. Results of E-Nose and E-Tongue

**Figure 6:**
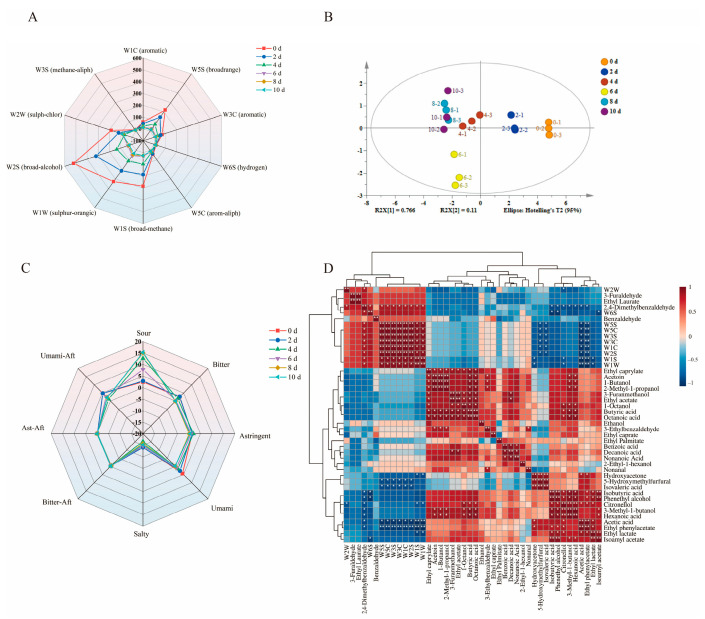
(**A**) Radar plot from the E-nose, (**B**) PLS-DA results from the E-nose dataset, (**C**) radar plot from the E-tongue, and (**D**) heatmap illustrating correlations between E-nose signals and volatile flavor compounds (* *p* < 0.05, ** *p* < 0.01). Red indicates positive correlations and blue denotes negative correlations.

The results of the E-nose analysis (Figure 6A) demonstrate a significant separation among samples from days 0, 2, 4, and 6, whereas the radar fingerprints of the electronic nose show an overlap between days 8 and 10. PLS-DA (Figure 6B) further confirmed distinct variations in the flavor profiles of BPV samples across different fermentation stages, showing clear segregation into separate regions. However, after day 8, the samples clustered together, consistent with the trends in the radar plot. This suggests that distinct fermentation stages lead to pronounced changes in aroma, which gradually stabilize as fermentation progresses. These findings are consistent with the analysis in Section 3.4. The variations in aroma profiles were driven by chemical transformations and microbial metabolic activities during fermentation [7]. The radar plot from the E-tongue (Figure 6C) indicates that the taste changes during the process can be divided into three phases: Phase I (0–4 days), Phase II (6 days), and Phase III (8–10 days). This is consistent with the analysis in Section 3.5. In the initial phase, the acidity increases, accompanied by a rise in bitterness and astringency, while the umami taste rapidly declines. Subsequently, the acidity continues to increase, but bitterness and astringency significantly decrease, and the aftertaste begins to converge. In the final stage, the taste gradually stabilizes with acidity as the core, accompanied by a slight bitterness and astringency, resulting in a more harmonious aftertaste. In fact, high concentrations of acetic acid can inhibit the perception and release of volatile flavor compounds [43], such as isoamyl acetate [44], which imparts a fruity aroma to vinegar.

Figure 6D demonstrates the correlation between E-nose data and volatile flavor compounds, with W1C and W5C clustering with acids and esters. Esters, alcohols, and aldehydes exhibited a strong correlation, clustering with W2W, W6S, and W3S. These findings suggest that the E-nose is highly sensitive to flavor compounds during fermentation, with acids, esters, and aldehydes playing a crucial role in the aroma profile of BPV. Acids contribute fatty, pungent, rancid, and fruity notes [11], esters serve as primary contributors to fruity and floral aromas [41], and aldehydes impart characteristic fruity and cream flavors to BPV [10]. Notably, volatile flavor compounds such as 2,4-dimethylbenzaldehyde (r = 0.95) and ethanol (r = 0.92) showed a strong correlation with W1S, while ethyl phenylacetate (r = 0.95) and acetic acid (r = 0.94) demonstrated a strong correlation with W5S. These two sensors exhibit sensitivity to aromatic compounds.

### 3.7. Correlation Between Volatile Flavor Compounds and Nonvolatile Flavor Compounds

**Figure 7:**
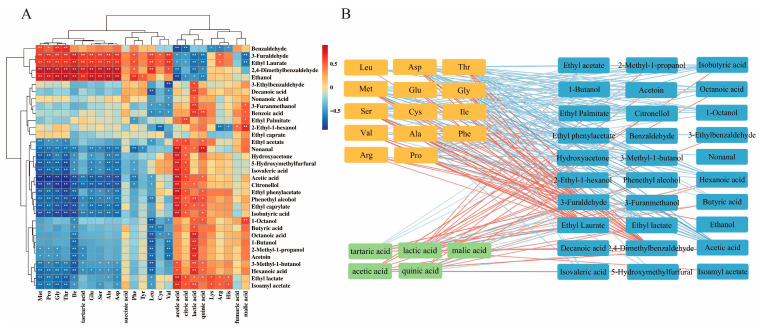
Correlation analysis between nonvolatile aroma compounds and volatile aroma compounds during BPV fermentation. Results show (**A**) association heatmap of Pearson analysis (* *p* < 0.05, ** *p* < 0.01), and (**B**) correlation network constructed by Spearman’s correlation coefficient (|r| > 0.6, *p* < 0.05). Red indicates positive correlations and blue denotes negative correlations.

Interactions among flavor compounds are likely to exist, and correlation analysis serves as a valuable tool for elucidating the potential relationships between volatile flavor compounds and substrates [12]. As shown in Figure 7A, most organic acids exhibited significantly positive correlations with volatile flavor compounds. Volatile compounds such as ethyl phenylacetate (r = 0.81), nonanal (r = 0.78), and ethyl caprylate (r = 0.76) exhibited a positive correlation with acetic acid. Similarly, 2-methyl-1-propanol (r = 0.7), acetoin (r = 0.7), and phenethyl alcohol (r = 0.7) showed a positive correlation with lactic acid. In addition, 2,4-dimethylbenzaldehyde (r = 0.76) and ethyl laurate (r = 0.63) were positively correlated with tartaric acid. It has been demonstrated that adding specific concentrations of organic acids enhances the release of volatile flavor compounds [45]. Elevated levels of lactic acid promote the volatilization of both ethyl lactate and ethyl acetate [46]. Therefore, organic acids influence the development of the volatile flavor profile, suggesting that their modulation may serve as a strategy for optimizing the flavor quality of vinegar.

Conversely, most volatile flavor substances demonstrated negative correlations with amino acids (Figure 7A). Notably, Met, Pro, Gly, Thr, Glu, Ser, Ala, Asp, Leu, Arg, Phe, Cys, Val, and Ile exhibited significant correlations with volatile flavor compounds. Among these, Met, Pro, Gly, Thr, and Leu showed particularly strong correlations with alcohols and esters, including citronellol, phenethyl alcohol, 3-methyl-1-butanol, ethyl phenylacetate, ethyl caprylate, isoamyl acetate, and ethyl lactate. For instance, a significant negative correlation was found between citronellol (r = −0.85), isobutyric acid (r = −0.78), and ethyl phenylacetate (r = −0.73) with Met. Similarly, citronellol (r = −0.76), isobutyric acid (r = −0.75), isovaleric acid (r = −0.70), and 5-hydroxymethylfurfural (r = −0.70) exhibited a significant negative correlation with Pro. Additionally, ethyl caprylate (r = −0.77) and phenethyl alcohol (r = −0.72) showed a negative correlation with Gly. Therefore, these compounds are hypothesized to participate in the biosynthesis of alcohols and esters, which collectively impart the fruity and floral aroma characteristics of BPV. Amino acids exhibit diverse roles in flavor development, functioning as precursors or intermediates that contribute directly or indirectly to the formation of aroma compounds [47], such as aldehydes, which are often derived from amino acid degradation [42]. Moreover, amino acids can modulate sensory perception by enhancing, altering, or masking specific flavor notes [31]. For example, alanine has been shown to attenuate the salty and bitter characteristics of vinegar [16]. During fermentation, amino acids are metabolized into their corresponding volatile flavor compounds through enzymatic degradation and Strecker degradation, thereby reducing the content of amino acids in the product [9]. This may explain the inverse correlation between free amino acids and volatile flavor compounds over time.

Figure 7B displays the correlation network (|r| > 0.6, *p* < 0.05) between volatile and nonvolatile flavor compounds. A total of 176 edges were identified (Supplementary 2), including 68 positive correlations (26 linked to organic acids, 42 to amino acids) and 108 negative correlations (12 associated with acids, 96 with amino acids). This result indicates that most volatile flavor compounds are negatively correlated with free amino acids, which is consistent with the observations in Figure 7A. These findings suggest that amino acids not only serve as key flavor-active components in BPV but also act as potential precursors to its volatile flavor compounds. Organic acids promote the release of volatile flavor compounds, but this effect is largely dependent on the acid concentration [45]. Such interactions are considered crucial for the aroma development of BPV. Targeted supplementation of these nonvolatile flavor compounds may offer a potential strategy to enhance the development of aroma during the fermentation process of BPV.

## 4. Conclusions

The acetic acid fermentation process of BPV enhances its functionality by enriching bioactive compounds. The results indicate a continuous increase in both TPC and TFC during the fermentation of BPV, while polysaccharides exhibited an initial decline followed by a gradual rebound. Moreover, fermentation markedly reduced 5-HMF levels, thereby diminishing constituents that may compromise product quality. A comprehensive and systematic investigation was conducted using GC-MS, HPLC, E-nose and E-tongue coupled with multivariate statistical analysis to characterize the temporal evolution and correlations between nonvolatile components (organic acids and free amino acids) and volatile flavor compounds during the fermentation of BPV. Notably, three volatile flavor compounds (isoamyl acetate, benzaldehyde, and nonanal) were distinguished not only as critical volatile flavor compounds (OAV > 1) but also as differential indicators (VIP > 1, *p* < 0.05). Throughout the fermentation process, the organic acid concentration gradually increases, while the amino acid content decreases. Correlation analysis confirmed that amino acids likely serve as aroma precursors for volatile flavor compounds during fermentation. The flavor profile of BPV undergoes pronounced dynamic changes throughout fermentation, with aromatic characteristics exhibiting progressive stabilization and reaching a relatively steady state by day 8. These insights provide a scientific basis for the development of blackened fruit-derived products and the directional regulation of flavor by free amino acids. In the future, further research will be conducted to explore the mechanisms of action of specific bioactive compounds, the dynamics and functional associations of microbial communities, as well as the targeted modulation of flavor by precursor substances at specific concentrations.

## Figures and Tables

**Table 1:** Ten sensors and their main applications of E-nose.

Sensor Name	Main Applications	Reference Material
W1W	Sensitive to sulfides compounds	H_2_S, 1 mg kg^−1^
W1C	Sensitive to aromatic compounds	Toluene, 10 mg kg^−1^
W3C	Sensitive to ammonia and aromatic compounds	Benzene, 10 mg kg^−1^
W6S	Mainly sensitive to hydrogen	H_2_, 100 µg kg^−1^
W5C	Sensitive to alkenes and aromatic compounds	Propane, 1 mg kg^−1^
W3S	Mainly sensitive to alkenes	CH_3_, 10 CH_3_, 100 mg kg^−1^
W1S	Sensitive to methane	CH_3_, 100 mg kg^−1^
W2S	Sensitive to alcohols, partially aromatic compounds	CO, 100 mg kg^−1^
W5S	Broad sensitivity and very sensitive to nitrogen oxides	NO_2_, 1 mg kg^−1^
W2W	Sensitive to aromatic compounds and organic sulfides	H_2_S, 1 mg kg^−1^

## Data Availability

The original contributions presented in this study are included in the article/Supplementary Material. Further inquiries can be directed to the corresponding author.

## Supplementary Materials

The following supporting information can be downloaded at: https://www.mdpi.com/article/10.3390/foods14162905/s1, Table S1: Analysis of physicochemical properties; Table S2: The changes of organic acids during different fermentation of BPV; Table S3: The highest proportion of organic acids during the fermentation process of BPV; Table S4: The changes in free amino acids during different fermentation of BPV; Table S5: Volatile compounds (µg/L) at different fermentation stages during acetic acid fermentation; Table S6: Odor activity values (OAV ≥ 1) of volatile flavor compound detected in the production of BPV. References [9,10,48,49,50,51] are cited in the supplementary materials.
