# Supplementary material for: Investigation on Precursor Aromas and Volatile Compounds During the Fermentation of Blackened Pear Vinegar
Source: Foods. 2025 Aug 21;14(16):2905. doi: 10.3390/foods14162905 (PMC12385226; doi:10.3390/foods14162905)
Supplement: Supplementary file 1 [file foods-14-02905-s001.zip › Supplementary1.pdf]

Supporting Information

Investigation on Precursor Aromas and Volatile Compounds during the Fermentation of Blackened Pear Vinegar

Shangjing Chen [1,†], Yuxiao Wang [1,†], Xin Sun [1], Zhizhen Han [2,3], Qiyong Jiang [1], Lin Gao [4], and Rentang Zhang [1,3,4,*]

1 College of Food Science and Engineering, Shandong Agricultural University, Tai'an 271018, China

2 Management Service Center of Laoling Agricultural Hi-Tech Industries Demonstration Zone, Dezhou 253600, China

3 Laoling Healthy Food Industry Technology Research Institute, Dezhou 253600, China

4 Laoling Tailetang Food Technology Co., Ltd., Dezhou 253600, China

* Correspondence: rentangzhang@163.com

† The authors contributed equally to this work.

Table S1 Analysis of physicochemical properties

	0 d	2 d	4 d	6 d	8 d	10 d
TPC (mg GAE/mL)	2.53±0.16c	2.72±0.09c	2.89±0.12c	3.23±0.21bc	3.77±0.60ab	4.26±0.63a
TFC (mg RE/100 mL)	8.17±2.11d	9.95±2.72d	17.94±1.83c	22.21±1.057b	23.43±1.83b	29.94±2.29a
Polysaccharides (mg/mL)	26.34±1.48de	21.58±5.05e	30.70±2.40cd	35.13±1.29bc	39.11±2.04ab	41.56±2.07a
5-HMF (mg/mL)	0.2003±0.0371a	0.0701±0.0115b	0.0037±0.0006c	0.0028±0.0003c	0.0029±0.0003c	0.0025±0.0002c

Notes: Values are means ±SD (n=3). Different letters in the same row indicate significant differences ($p < 0.05$).

Table S2 The changes of organic acids during different fermentation of BPV.

Organic acid	Concentration(g/L)					
	0 d	2 d	4 d	6 d	8 d	10 d
tartaric acid	0.96±0.06a	0.69±0.04b	0.5±0.08bc	0.56±0.07bc	0.65±0.21bc	0.46±0.06c
malic acid	0.64±0.18b	2.54±1.37a	2.08±0.27a	1.73±0.29ab	1.91±0.71ab	1.3±0.18ab
lactic acid	0.43±0.18c	1.33±0.22b	1.55±0.53ab	1.98±0.24a	1.48±0.33ab	1.32±0.17b
acetic acid	0.42±0.25c	0.51±0.07c	0.53±0.2c	1.36±0.53bc	2.28±1.12ab	3.11±1.06a
citric acid	0.33±0.03b	0.62±0.4ab	0.32±0.08ab	0.59±0.02ab	0.64±0.22ab	1.07±0.39a
succinic acid	0.35±0.1a	0.26±0.02ab	0.16±0.04b	0.35±0.05a	0.26±0.09ab	0.24±0.06ab
fumaric acid	0.1±0.09	0.17±0.06	0.13±0.03	0.11±0.02	0.12±0.03	0.11±0.01
quinic acid	1.58±0.09b	2.6±0.5ab	2.38±0.74ab	2.67±0.43a	2.97±0.62a	2.64±0.23a
Total eight organic acids	4.82±0.53b	8.71±1.3a	7.66±1.76ab	9.35±0.77a	10.29±2.38a	10.24±1.04a

Notes: Values are means ± SD (n = 3). Means in the same line followed by different letters are significantly different (p <0.05).

Table S3 The highest proportion of organic acids during the fermentation process of BPV.

Fermentation time	Proportion (%)							
	tartaric acid	malic acid	lactic acid	acetic acid	citric acid	succinic acid	fumaric acid	quinic acid
8 d	6.3%	18.6%	14.3%	22.1%	6.2%	2.5%	1.1%	28.9%

Table S4 The changes in free amino acids during different fermentation of BPV.

Free amino acid	Taste attributes	Concentration (mg/L)					
		0 d	2 d	4 d	6 d	8 d	10 d
Asp	umami	175.76±84.14a	102.68±26.84b	14.43±9.12c	1.68±1.56c	2.36±0.78c	1.13±0.27c
Glu	umami	14.31±0.50a	8.64±0.98bc	8.60±5.05bc	10.60±4.84ab	7.23±0.82bc	5.80±0.76c
Total umami		190.07±84.29a	111.31±27.53b	23.03±12.14c	12.27±3.47c	9.59±1.27c	6.93±0.77c
Gly	Sweet	4.13±0.61a	2.17±1.28b	0.74±0.64c	0.43±0.36c	0.78±0.30c	0.27±0.17c
Ala	Sweet	29.76±1.25a	15.13±0.88b	4.62±4.03c	8.24±5.15cd	4.88±2.12cd	0.67±0.16c
Thr*	Sweet	218.12±24.50ab	326.32±475.90a	32.53±6.94ab	9.27±4.72b	5.77±1.55b	4.29±2.05b
Ser	Sweet	19.41±2.39a	4.76±4.39b	0.81±0.95c	1.05±0.96c	1.99±1.03bc	1.86±0.61bc
Pro	Sweet	6.34±1.46a	3.05±1.14ab	0.47±0.27b	4.66±7.95ab	NF	NF
Total sweet		277.76±27.12ab	351.43±473.30a	39.17±7.48b	23.66±11.52b	13.43±4.02b	7.09±2.38
Met*	bitter	10.43±1.38a	4.11±3.89b	0.21±0.36c	0.65±0.44c	NF	NF
Ile*	bitter	7.55±4.27a	2.06±1.90b	2.08±4.52b	1.38±1.33b	NF	NF
Leu*	bitter	15.99±0.74a	13.16±2.68ab	8.26±3.56bcd	7.57±4.24cd	6.42±4.63d	11.86±0.65abc
Phe*	bitter	7.36±0.47ab	3.51±1.58b	6.14±4.92ab	4.18±1.77ab	4.06±1.33ab	2.06±0.25b
Lys*	bitter	1.39±0.10bc	1.22±0.23bc	0.22±0.13d	1.68±0.58ab	2.32±1.01a	0.82±0.36cd
His	bitter	0.90±0.05a	0.21±0.09b	0.81±1.02bc	1.32±0.70a	0.78±0.26ab	0.40±0.15b
Arg	bitter	1.65±0.16a	NF	NF	1.37±1.04a	1.35±0.53a	0.44±0.09b
Tyr	bitter	5.23±0.44ab	3.47±1.30b	9.87±10.99a	5.28±1.05ab	4.76±0.58ab	4.16±0.22ab
Val*	bitter	41.82±4.48a	25.88±2.37bc	13.80±6.52d	21.66±1.07c	30.49±1.48b	25.43±1.37bc
Total bitter		92.32±7.75a	53.63±8.95b	37.38±6.06c	45.09±6.80bc	50.18±4.47b	45.17±1.30bc
Cys	Tasteless	1.01±0.10a	0.04±0.07b	0.04±0.07b	0.24±0.37b	NF	0.97±0.60a
Content of total free amino acids		561.16±116.21a	516.41±452.37a	99.62±20.55b	81.24±18.21b	73.20±7.81b	60.16±2.46b

Notes: Each value was expressed as mean ± SD (n = 5). NF, not detected. Values followed by different letters in the same row are significantly different ($p < 0.05$). *Essential amino acids

Table S5 Volatile compounds (μg/L) at different fermentation stages during acetic acid fermentation

Compound	0d	2d	4d	6d	8d	10d
Alcohols						
Ethanol	6452.31±1285.54a	4899.04±632.09b	4262.25±561.84b	3894.29±649.59bc	3678.21±430.21bc	2432.45±704.7c
3-Methyl-1-butanol	4.71±0.56d	78.07±68.64cd	407.8±139.42b	829.65±289.1a	554.33±107.8ab	341.47±15.36bc
Phenethyl alcohol	10.04±6.11d	26.33±9.96d	152.53±33.19cd	398.45±148.77a	340.05±79.07ab	224.07±75.91bc
2-Methyl-1-propanol	NF	34.61±6.96cd	106.26±54.59b	204.73±48.62a	98.39±30.82bc	33.99±13.42cd
1-Butanol	NF	2.67±1.08c	6.45±1.36b	10.34±3.33a	6.29±1.59b	2.45±1.42a
1-Octanol	NF	3.48±0.29ab	5.08±1.76ab	5.79±1.58a	5.9±3.00a	2.21±1.17bc
2-Ethyl-1-hexanol	NF	26.66±1a	14.45±3.16b	10.35±0.73c	12.2±1.44bc	4.67±0.16d
Total	6467.39±1291.03a	5070.87±574.59ab	4954.82±720.1b	5353.6±245.35ab	4695.35±309.44b	3041.3±780.36c
Acids						
Acetic acid	562.35±89.67a	2423.67±1026.78a	5430.48±2209.71c	8444.63±1494.03b	12269.78±449.8a	11820.18±1100.11a
Isobutyric acid	NF	5.04±8.72b	21.18±6.72b	52.65±26.98a	55.6±16.94a	54.83±0.4a
Decanoic acid	2.78±2.99b	9.4±16.27ab	11.95±5.13ab	19.32±4.29a	12.2±1.88ab	3.21±2.98ab
Nonanoic Acid	4.79±5.52b	11.55±5.04ab	9.82±2.95ab	15.2±3.88a	9.34±4.31ab	5.49±0.39b
Isovaleric acid	NF	5.04±8.72b	21.18±6.72b	52.65±26.98a	55.6±16.94a	54.83±0.4a
Hexanoic acid	1.16±0.59b	5.36±0.14b	9.39±2.84b	21.37±8.07a	20.33±9.17a	7.27±2.02b
Octanoic acid	2.96±1.87d	7.15±1.44cd	9.2±0.59bc	15.86±3.8a	15.01±5.71ab	5.77±0.86cd
Butyric acid	NF	2±0.81bc	3.27±0.54bc	7.28±2.28a	4.35±1.77ab	1.64±2.84bc
Benzoic acid	0.64±0.25c	9.63±7.04a	2.45±0.43bc	7.72±1.67ab	3.88±1.62abc	2.23±0.45bc
Total	574.67±96.81d	2478.83±992.07d	5518.92±2211.29c	8636.68±1446.04b	12446.09±441.64a	11955.45±1095.56a
Esters						
Ethyl acetate	NF	1472.27±209.45ab	959.08±9.50ab	2226.89±1900.62a	2024.64±299.44a	1243.42±318.87ab

Ethyl caprate	25.84±15.07b	31.34±5.1b	57.75±5.93a	56.65±5.87a	26.53±5.8b	29.55±4.58b
Ethyl caprylate	8.8±3.07c	8.95±0c	15.07±3.46bc	55.37±28.88a	35.1±3.32ab	33.66±11.05abc
Ethyl phenylacetate	NF	NF	0.29±0.5b	23.48±7.47a	32.09±7.25a	24.75±4.84a
Ethyl lactate	NF	NF	NF	19.9±6.76a	22.47±0.05a	10.1±1.11b
Isoamyl acetate	NF	NF	NF	81.03±10.97a	13.11±0.05b	10.92±2.63b
Ethyl laurate	23.68±11.5a	NF	NF	NF	NF	NF
Ethyl Palmitate	6.24±3.23a	19.28±4.79b	6.61±3.43a	15.22±2.88a	12.92±6.54ab	15.33±3.08a
Total	64.56±31.59b	1531.84±208.14ab	1038.4±17.77ab	2478.21±1917.67a	2166.86±316.62a	1367.73±305.36ab
Aldehydes and Ketones						
Acetoin	NF	34.61±6.96cd	106.26±54.59b	204.73±48.62a	98.39±30.82bc	33.99±13.42cd
5-Hydroxymethylfurfural	NF	NF	NF	NF	37.17±0.01a	45.4±18.21a
3-Furaldehyde	102.48±39.44a	NF	NF	NF	NF	NF
2,4-Dimethylbenzaldehyde	14.13±6.88a	6.34±0b	NF	NF	NF	NF
Benzaldehyde	16.18±3.06b	17.99±1.9b	33.52±1.51a	NF	NF	NF
3-Ethylbenzaldehyde	NF	NF	2.51±0.82a	2.77±0.66a	NF	NF
Nonanal	0.87±0.11a	6.38±1.39b	6.34±0.89b	6.84±0.63b	7.23±0.64b	7.98±1.11b
Total	133.65±46.78bc	65.32±6.57c	148.62±53.17ab	214.34±49.53a	142.79±31.12abc	87.37±30.53bc
Others						
Hydroxyacetone	NF	NF	NF	NF	6.07±0.67a	3.3±1.25b

3-Furanmethanol	0.86±0.36c	11.69±2.48ab	11.1±4.76b	19.87±7.60a	12.72±4.32ab	4.76±3.21bc
Citronellol	NF	NF	15.05±1.85b	42.87±5.16a	42.71±2.14a	14.36±3.02b
Total	0.86±0.36d	11.69±2.48c	26.15±7.85b	62.74±5.56a	61.51±4.64a	22.42±0.74b

Note: Data represent the means ± SD (n = 3). Different letters represent significant difference in the same line ($p < 0.05$). NF, not found.

Table S6 Odor activity values (OAV ⩾ 1) of volatile flavor compound detected in the production of BPV.

volatile compounds	Threshold (μg/L)[a]	Description	0 d	2 d	4 d	6 d	8 d	10 d	Ref[b]
3-Methyl-1-butanol	250	alcohol, chemical	0.02	0.31	1.63	3.32	2.22	1.10	c
Phenethyl alcohol	390	rose, sweet	0.026	0.068	0.39	1.02	0.87	0.57	d
Ethyl caprate	53.2	fruity, fatty, pleasant	0.49	0.59	1.09	1.06	0.50	0.56	e
Ethyl caprylate	5	sweet, soapy, apple	1.76	1.79	3.01	11.07	7.02	6.73	f
Isoamyl acetate	2	banana, fresh, pear	0	0	0	40.51	6.56	5.46	g
Citronellol	40	roses, peaches, citrus	0	0	0.38	1.07	1.07	0.36	h
Benzaldehyde	14	green, fatty, lavender	1.16	1.29	2.39	0	0	0	g
Nonanal	1.1	almond	0.79	5.79	5.76	6.22	6.57	7.25	f

a Odor thresholds in water taken from references in the literature.

b References of odor threshold.

c [9]

d [10]

e [48]

f [49]

g [50]

h [51]

References

9. Tian, H.; Xiong, J.; Sun, J.; Du, F.; Xu, G.; Yu, H.; Chen, C.; Lou, X. Dynamic transformation in flavor during hawthorn wine fermentation: Sensory properties and profiles of nonvolatile and volatile aroma compounds coupled with multivariate analysis. *Food Chem.* **2024**, *456*. http://doi.org/10.1016/j.foodchem.2024.139982.
10. Zhang, L.; Qin, Z.; Zhang, L.; Jiang, Y.; Zhu, J. Dynamic changes of quality and flavor characterization of Zhejiang rosy vinegar during fermentation and aging based on untargeted metabolomics. *Food Chem.* **2023**, *404*. http://doi.org/10.1016/j.foodchem.2022.134702.
48. Benkwitz, F.; Nicolau, L.; Lund, C.; Beresford, M.; Wohlers, M.; Kilmartin, P.A. Evaluation of Key Odorants in Sauvignon Blanc Wines Using Three Different Methodologies. *J. Agr. Food Chem.* **2012**, *60*, 6293-6302. http://doi.org/10.1021/jf300914n.
49. Feng, Z.; Martínez-Lapuente, L.; Ayestarán, B.; Guadalupe, Z. Volatile and sensory characterization of Tempranillo wines aged in *Quercus alba* oak barrels of different geographical origins in USA. *LWT* **2023**, *173*. http://doi.org/10.1016/j.lwt.2022.114328.
50. Van Gemert, L.J. Compilations of odour threshold values in air, water and other media *(2th ed.)*. Oliemans Punter & Partners BV, Zeist: 2011.
51. Delgado, J.A.; Sanchez-Palomo, E.; Alises, M.O.; Vinas, M.A.G. Chemical and sensory aroma typicity of La Mancha Petit Verdot wines. *LWT* **2022**, *162*. http://doi.org/10.1016/j.lwt.2022.113418.
